# Combination of Alcohol and Fructose Exacerbates Metabolic Imbalance in Terms of Hepatic Damage, Dyslipidemia, and Insulin Resistance in Rats

**DOI:** 10.1371/journal.pone.0104220

**Published:** 2014-08-07

**Authors:** Salamah Mohammad Alwahsh, Min Xu, Frank Christian Schultze, Jörg Wilting, Sabine Mihm, Dirk Raddatz, Giuliano Ramadori

**Affiliations:** 1 Department Gastroenterology and Endocrinology, University Medical Center Goettingen, Goettingen, Germany; 2 Department of General, Visceral, and Pediatric Surgery, University Medical Center Goettingen, Goettingen, Germany; 3 Institute of Anatomy and Cell Biology, University Medical Center Goettingen, Goettingen, Germany; Institute of Medical Research A Lanari-IDIM, University of Buenos Aires-National Council of Scientific and Technological Research (CONICET), Argentina

## Abstract

Although both alcohol and fructose are particularly steatogenic, their long-term effect in the development of a metabolic syndrome has not been studied *in vivo*. Consumption of fructose generally leads to obesity, whereas ethanol can induce liver damage in the absence of overweight. Here, Sprague-Dawley rats were fed *ad libitum* for 28 days on five diets: chow (control), liquid Lieber-DeCarli (LDC) diet, LDC +30%J of ethanol (L-Et) or fructose (L-Fr), and LDC combined with 30%J ethanol and 30%J fructose (L-EF). Body weight (BW) and liver weight (LW) were measured. Blood and liver samples were harvested and subjected to biochemical tests, histopathological examinations, and RT-PCR. Alcohol-containing diets substantially reduced the food intake and BW (≤3^rd^ week), whereas fructose-fed animals had higher LW than controls (*P*<0.05). Additionally, leukocytes, plasma AST and leptin levels were the highest in the fructose-administered rats. Compared to the chow and LDC diets, the L-EF diet significantly elevated blood glucose, insulin, and total-cholesterol levels (also *vs*. the L-Et group). The albumin and Quick-test levels were the lowest, whereas ALT activity was the highest in the L-EF group. Moreover, the L-EF diet aggravated plasma triglyceride and reduced HDL-cholesterol levels more than 2.7-fold compared to the sum of the effects of the L-Et and L-Fr diets. The decreased hepatic insulin clearance in the L-EF group *vs*. control and LDC groups was reflected by a significantly decreased C-peptide:insulin ratio. All diets except the control caused hepatosteatosis, as evidenced by Nile red and H&E staining. Hepatic transcription of insulin receptor substrate-1/2 was mainly suppressed by the L-Fr and L-EF diets. The L-EF diet did not enhance the mitochondrial *β-*oxidation of fatty acids (*Cpt1α* and *Ppar-α* expressions) compared to the L-Et or L-Fr diet. Together, our data provide evidence for the coaction of ethanol and fructose with a high-fat-diet on dyslipidemia and insulin resistance-accompanied liver damage.

## Introduction

Currently, fast-food meals, which are characterized by standardized palatability, high contents of fat and sugar, large portion sizes and high energy densities [1], are frequently consumed with alcoholic beverages. The number of fast food outlets and sales has increased over the past 30 years due to aspects of convenience, such as time-saving, minimal waiting, easy purchasing through self-service or carry-out eating venues [2], [3]. Fast-food consumption is associated with weight gain and insulin resistance (IR) in humans [4]. Furthermore, feeding experimental animals with a “cafeteria diet” leads to liver damage [5].

Nutrients may have dual influences on the body, acting either as a benefit or risk for life sustainability, according to the type and amount of consumed dietary ingredients. The consumption of fructose beverages favors the increasing prevalence of metabolic syndrome and such accompanying diseases as nonalcoholic fatty liver disease (NAFLD) [6]. NAFLD is defined as the accumulation of lipid in the liver of individuals who do not consume significant amounts of alcohol (30–40 g/day) [7] and in whom other known causes of steatosis, such as certain drugs and toxins, have been excluded [8]. NAFLD which can progress to liver cancer, is a multifactorial disease that involves complex processes of the genetics, diet, and lifestyle [9]. The pathogenesis of NAFLD and the efficacy and safety of its pharmacotherapy remain elusive [10].

The Western-style diet is often enriched in both, saturated fat and sugar. The total caloric intake plays an important role in the development of NAFLD [11]. In the United States, the ingestion of fructose has increased by approximately fivefold between 1975 and 2000 [12]. Fructose, a fruit sugar, is commonly used as a sweetener, e.g., in high-fructose corn syrup [13]. It is frequently found in soft drinks and juice beverages and can be integrated into convenient, pre-packaged food. The term “soft drink” encompasses *sodas* (carbonated beverages, e.g., Colas) and other sugar-sweetened beverages such as fruit drinks and lemonade [14]. As fructose has been demonstrated to induce NAFLD in both humans [7]and animals [15], [16]; it should be studied more closely.

In addition to fructose-enriched meals, alcoholic (ethanolic) beverages are also consumed regularly by a large proportion of the population. In many countries, light-to-moderate alcohol consumption is considered to be an integral part of the diet [17]. Beer is the world’s most widely consumed alcoholic beverage and generally contains 4–6% (*v/v*) alcohol [18]. The amount of ethanol consumed determines its role in the nutritional balance; therefore, alcohol abuse leads to alcoholic fatty liver disease (AFLD) [19]. However, the effects of light alcohol consumption (e.g., <140 g/week) [20] on NAFLD have not been well documented, and in particular, its combination with a (high) fructose diet has not been determined.

As people consume various types of food, we sought to develop and characterize models of fatty liver based on nutritional compositions resembling those of fast food, Western-style diets and alcoholic beverages, using a multipurpose outbred rat stock. We experimentally compared the effects of selected nutrients, their relative contribution and interaction, and metabolic regulatory pathways on the development of hepatic damage and insulin resistance, and metabolic syndrome.

## Materials and Methods

### Animals

Ten-week old male Sprague-Dawley rats (270–310 g) were purchased from Charles River, Sulzfeld, Germany. The rats were housed individually at 24°C in conventional cages under a 12∶12 h light-dark schedule. All animals received humane care according to the criteria outlined in the “Guide for the Care and Use of Laboratory Animals” by the German National Institutes of Health. The experiments were ethically approved by authorities and guidelines of Georg-August-University of Goettingen.

### Experimental design

**Figure 1:**
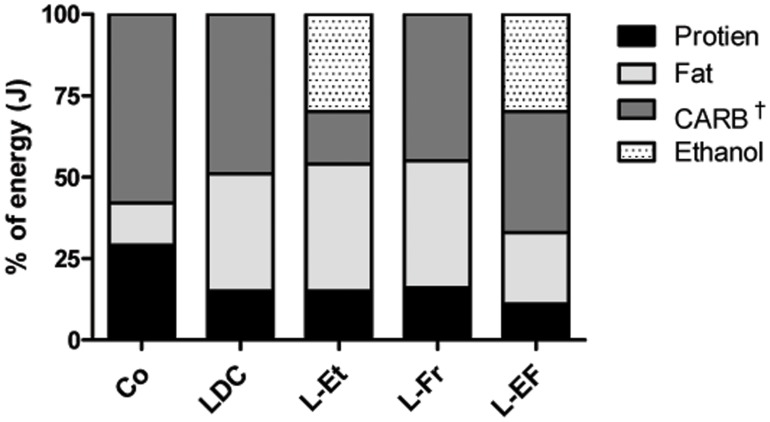
Nutrient profiles of the rat’ feeding categories. Stacked columns show the percentage of energy contribution of the following macronutrients: protein, fat, and carbohydrates (CARB), as well as ethanol, in relevant diet category. The standard chow (Co) diet contained 58% CARB-derived calories (mainly starch). The Lieber-DeCarli (LDC) diet contained 49% calories CARB (mainly maltodextrin). In contrast, the L-Et was composed of 16% CARB-derived calories including ethanol (30%). The L-Fr diet included 45% CARB-derived calories (30% fructose). The L-EF diet contained 37% CARB (30% fructose), and 30% ethanol-derived energy. ^†^indicates that each of L-Fr and L-EF diets implicates 30% J fructose, whereas in the other groups, the energy generated from fructose is negligible.

The rats were randomly assigned to one of five groups (4–6 per group), according to the following diets: (1) Control group, which consumed commercially pelleted chow; (2) Liquid Lieber-DeCarli (LDC) group [15]; (3) LDC +30% J (4.7% w/v) ethanol (L-Et); (4) LDC +30% J fructose (L-Fr); and (5) LDC paired with 30% of energy derived from ethanol and 30% from fructose (L-EF) group (Fig. 1). The LDC diet (Table S1) was provided as a powder by ssniff Spezialdiäten, Soest, Germany. The fat-derived energy was approximately the same in the LDC, L-Et, and L-Fr, and similar diets with a dietary content of 36–39% fat-derived energy are considered to be high-fat diets (HFD) [21], [22]. The L-Fr diet is also known as a Western diet because it consists of high fructose HFD [21]. One kg of each absolute liquid diet was adjusted to contain ≈4.184 kJ (1 kcal). The animals received their adequate pre-weighed food *ad libitum* for 28 days (after 5 days of dietary adaptation). The amounts of consumed food were measured daily by subtracting the pre-weighted food from the remaining food. The animals’ body weights (BW) were measured at the beginning of the experiment and then weekly throughout the study. In addition to *ad libitum* sampling, subgroups (4 rats per each) were reared on the chow, LDC, or L-EF diet, and then were overnight fasted before sacrifice.

### Harvesting of liver and blood samples

The rats were weighed and anesthetized intraperitoneally with sodium pentobarbital (Merial, Hallbergmoos, Germany, 0.2 mL per 100 g BW) to reduce suffering. Blood samples were taken from the *inferior vena cava* into plain (serum), heparinized (plasma), EDTA (complete blood count), sodium citrate (coagulation assay) tubes. Subsequently, the livers were excised, weighed, and rinsed in physiological saline. Then, liver portions of each animal were fixed in 4% neutral-buffered formalin for paraffin embedding. Alternatively, liver pieces were flash-frozen in liquid nitrogen, and together with serum and plasma aliquots, were stored at −80°C. Relative liver weights (RLW, %) were expressed as a ratio of absolute liver weight (LW, g), divided by the total BW (g) at the time of sacrifice.

### Clinical chemistry assays

The heparinized blood was centrifuged at 3,500×*g* at 4°C for 15 min. Glucose levels, total lipid profile, uric acid, urea, bilirubin, electrolytes, iron, and the activities of alanine aminotransferase (ATL), aspartate aminotransferase (AST), and alkaline phosphatase (AP) were determined in the harvested plasma by utilizing automated systems in the Department of Clinical Chemistry of the UMG. In addition, serum leptin levels were measured with a radio-immuno-assay (RIA) kit (Millipore, MA, USA) for all groups. Serum C-peptide and insulin concentrations were evaluated in the Co, LDC, and L-EF groups with RIA (Millipore, MA, USA). The overnight-fasted animals were tested for plasma glucose and TG, and serum insulin and C-peptide levels.

### Determination of hepatic triglycerides (TG) and cholesterol

To quantify the hepatic TG and cholesterol content, fresh liver samples (about 100 mg) were homogenized in ice-cold 2× PBS using a tissue lyser. The lipids were extracted with 2∶1 (*v/v*) methanol:chloroform, vacuum dried, and resuspended in 5% *(w/v)* fat-free bovine serum albumin. TG levels were determined colorimetrically using a commercially available kit (BioAssay Systems, EnzyChrom Kit, USA). Values were normalized to the initial wet weight of the liver portion and expressed as mM TG or cholesterol/g liver.

### Histopathological studies of the liver

#### Nile red staining

Fluorescent Nile red dye, 9-diethylamino-5H-benzo[α]phenoxazine-5-one, is an excellent vital and selective stain for the detection of intracellular neutral lipid droplets by fluorescence microscopy [23]. Nile red does not fluoresce in aqueous or polar solutions; rather, it is easily soluble in hydrophobic phases and excites at λ 450–560 nm, with its emission detected at λ = 590 nm. This difference in the excitation and emission maxima of a fluorescent dye is called Stoke’s shift and represents the basis for fluorescence detection. To localize TG in the liver tissues, the samples were freshly collected and immediately frozen in liquid nitrogen. Nile red stock solution (10 µg/mL) was prepared in acetone and light-protected with aluminum foil at 4°C. The liver specimens were cut into 5-µm slices using a cryostat and placed on frosted slides. The hepatic sections were fixed with 4% (*v/v*) neutral-buffered formaldehyde for 10 min, followed by 5 min of washing. The slides were then stained with 4,6-diamidino-2-phenylindole (DAPI) for 5 min and washed for 5 min with PBS. A mixture of 750 µL of 65°C heated-Kaiser’s glycerol gelatin, 250 µL of distilled H_2_O and 50 µL of Nile red solution was added to every 4 slides. A mixture without dye solution was simultaneously added to a negative control slide. The sections were then cover-slipped, allowed to dry shortly in the dark, and studied with an epifluorescence microscope (Axiovert 200 M, Zeiss, Jena, Germany).

#### Haematoxylin and Eosin (H&E) staining

Liver pathological changes were evaluated by H&E staining. Paraffin-embedded specimens were cut into serial sections of 5-µm thickness with a microtome. Sections were deparaffinized in xylene, rehydrated through a graded ethanol series and stained automatically with H&E. After mounting with xylene-based media, the slides were histologically examined under a light microscope (Olympus BX43) equipped with an internal digital camera (Olympus DP21). The nuclei were stained blue-purple with Hematoxylin, while cells’ cytoplasms (proteins) were nonspecifically counterstained with pink-red (eosin). Coded H&E-stained liver sections were evaluated for inflammation and fibrosis by an experienced pathologist in a blinded manner. Fibrosis was diagnosed according to Desmet/Ishak (stage 0–4), with 0 =  no fibrosis, 1 =  minimal fibrosis, mild portal connective tissue, 2 =  mild fibrosis increased portal fibrosis with beginning extension into/affection of the hepatic lobule, 3 =  moderate portal connective tissue formation with complete and incomplete septa, and 4 =  cirrhosis. Inflammation was evaluated with grade 0–4 (no inflammation to necrosis), accordingly.

### RNA extraction, reverse transcription, and real-time PCR

To isolate total RNA, frozen liver samples were homogenized within 48 h after rats’ death in reagents of Qiagen RNeasy Mini Kit (Qiagen, Hilden, Germany). The samples were treated with RNase-free DNase, and the extracted RNA concentrations were determined. Next, 1 µg RNA was used for complementary DNA (cDNA) synthesis by reverse transcriptase (M-MLV RT). Subsequently, 6.3 ng cDNA was added to 9 µL mixture of targeted primer-pair and Fast Platinum SYBR Green Universal master mix (Applied Biosystems, life technologies, Germany). The following PCR primers were used: *Acc-2*: Fwd 5′ATGGTCATGCGTTACGGCA3′ and Rev 5′CGGACTCGTTGGTGATGAAGA3′, *Cpt1-α*: Fwd 5′CGGTTCAAGAATGGCATCATC3′ and Rev 5′TCACACCCACCACCACGAT3′, *Cd36*/*fat*: Fwd 5′CCATTCTTGAGTTTGGTTCCAT3′ and Rev 5′GCATCTGTGCCATTAATCATGT3′, *Hl*: Fwd 5′CCCTACAAAGTATTCCATTACC3′ and Rev 5′CCGTGTAAATCAAGAAGGAG3′, *Ppar-α*: Fwd 5′GTCATCACAGACACCCTCTC3′, and Rev 5′CAGCTTCGATCACACTTGTC3′, *Lep-r*: Fwd 5′GTTCTGGCCATCAATTCCAT3′ and Rev 5′GCCCTCTGGTTGCTTTGTAT3′, *Irs-1*: Fwd 5′CACAGAGAGTGGACCCCAAT3′ and Rev 5′GCTCTCAACAGGAGGTTTGG3′, *Irs-2*: Fwd 5′CAGTAGCCACAGGAGCAACA3′ and Rev 5′CAGGCGTGGTTAGGGAGTAA3′, *Actb*: Fwd 5′ACCACCATGTACCCAGGCATT3′ and Rev 5′CCACACAGAGTACTTGCGCTCA3′. *Ubc*: Fwd 5′CACCAAGAAGGTCAAACAGGAA3′, and Rev 5′AAGACACCTCCCCATCAAACC3′.

β-actin (*Actb*) and *Ubc* were designed as endogenous references (housekeeping genes). The amplification of a total of 10 µL/well was performed in duplicate through two-step cycling (95–60°C) for 40 cycles in a StepOne Plus quantitative real-time PCR detection system. The comparative C_T_ – method was used to determine the amount of target gene, normalized to the housekeeping gene and relative to a calibrator (ΔΔC_T_). All RT-PCR data are expressed as fold-change compared to data of the control group. A melting curve analysis was executed at the end of each PCR run, to confirm that only one PCR product was present.

### Statistical analysis

All statistical calculations and data graphics were performed with Microsoft Excel (unpaired t-test, when needed), and Graph Pad Prism 5 software (San Diego, CA) using ANOVA (Tukey’s post hoc test) to examine the statistical significance (*P*<0.05) amongst the means of all groups. Data are presented as mean ± SEM of 4–6 rats per group.

## Results

### Characteristics of the animals

The rats were longitudinally followed to assess their energy consumption and weight gain.

#### Food intake

**Figure 2:**
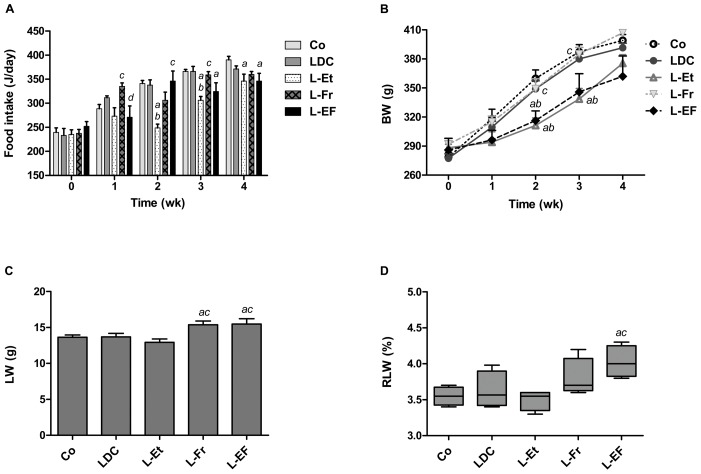
Animals’ phenotypes. Growth curves of rats fed the LDC, L-Et, L-Fr or L-EF diet (A); curves of food intake (B); bars show liver weights (C); and relative liver weights (D) after the rats had been fed with different diets *ad lib* for 28 d. The mean values are shown with SEMs represented by vertical bars for 6 rats per group. *^abcd^* designate that the mean values were statistically dissimilar differences from the corresponding group *vs*. Co, LDC, L-Et, or L-Fr groups, respectively. Co: chow, LDC: liquid Lieber-DeCarli diet, L-Et: LDC +30% J ethanol, L-Fr: LDC +30% J fructose, L-EF: LDC+combination of 30% J ethanol and 30% J fructose.

The basal daily average of energy consumption was 234 J per rat. There was no significant difference in the context of food intake between the control, LDC, or L-Fr groups throughout the study (Fig. 2A). After one week, food consumption by the L-Fr group was significantly higher compared to that of the L-Et and L-EF groups. In contrast, food intake was reduced in rats fed with ethanol-containing diets especially at the 3^rd^ and 4^th^ weeks (*P*<0.05), and fructose supplements (as a sweetener) did not encourage a greater intake in the L-EF group. No mortality occurred during the entire study.

#### Changes in the rats’ BW, LW, and RLW

Although the initial BW of the groups did not vary, supplementation with ethanol (in the L-Et and L-EF diets) generally decreased animals’ BW gain, which was more apparent during the 2^nd^ and 3^rd^ week of feeding compared to the other groups (Fig. 2B). The L-Et diet significantly reduced the BW compared to the chow, LDC, or L-Fr diet at the 2^nd^ and 3^rd^ week. At the end of the experiment, the overall weight gains of the rats were 117 g (control), 110 g (LDC), 81 g (L-Et; *P*<0.01 *vs*. control), 102 g (L-Fr), and 75 g (L-EF; *P*<0.01 *vs*. control). As shown in Fig. 2B, the BW curves of the control and LDC groups are quite similar with a strong progressive increase over the study time. In the L-Fr group, the addition of 30% J fructose to the LDC diet did not significantly influence the BW gain during this feeding period. The accelerated rat weight gain of the control, LDC, and L-Fr groups began to slow down after 3 weeks of the treatment. On the other hand, the BW of the L-Et and L-EF groups had almost been restored at week 4, although it was still lower than the other groups. Fructose-fed rats (diets L-Fr and L-EF) had higher LW compared to the L-Et- or chow-fed rats (Fig. 2C, *P*<0.05). Consequently, the RLW of the L-EF group was higher in comparison to that of the control and L-Et groups (Fig. 2D, *P*<0.05).

### Histology of liver tissues and evaluation of the intrahepatic lipids

**Figure 3:**
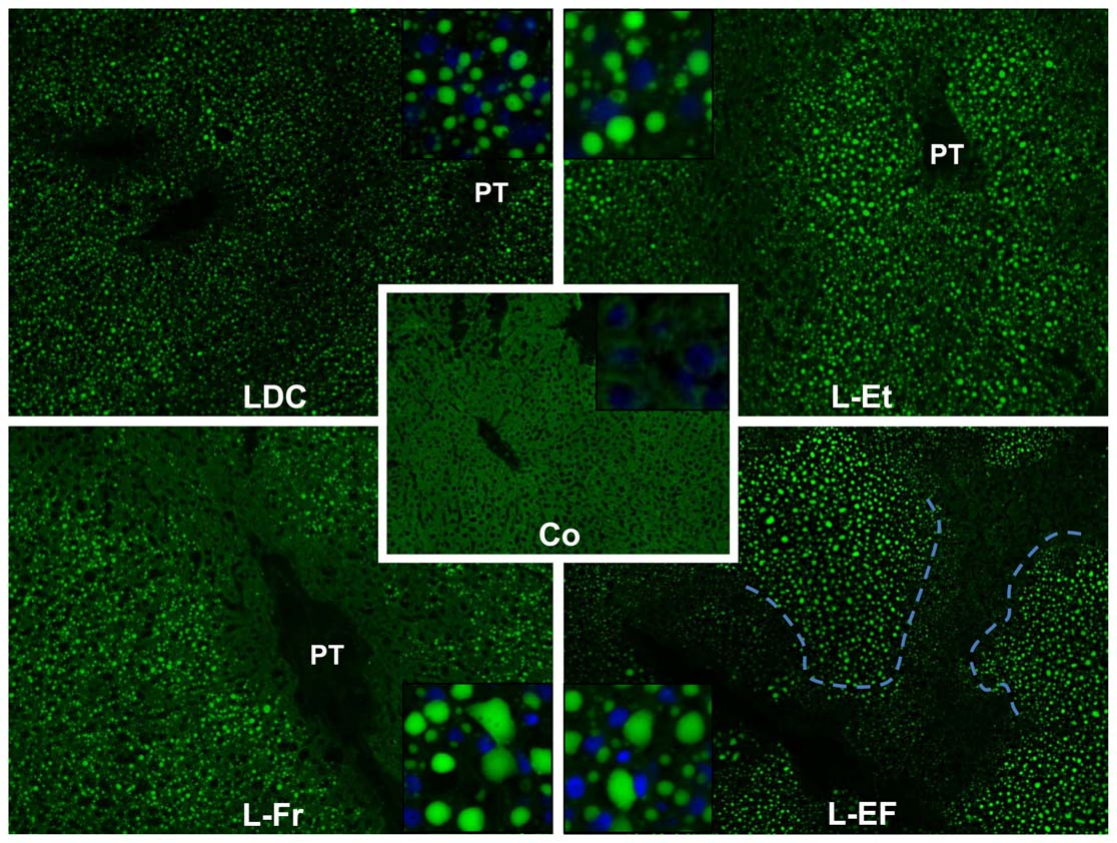
Representative photomicrographs of Nile red-stained liver sections. The stained TG are appeared as glittered, spherical, discrete droplets distributed throughout the cytoplasm (×100). The insets (×200) show hepatocytes with DAPI stained nuclei (blue) and fat droplets (green) in the experimental groups. A distinct border between zone I and the unaffected zone III was created by hepatocytes with tiny-droplet (microvesicular) steatosis in the L-EF group. PT: portal triad, Co: control, LDC: Lieber-DeCarli diet, L-Et: LDC +30% J ethanol, L-Fr: LDC +30% J fructose, L-EF: LDC+isoenergetic (30% J) ethanol and fructose.

To study the deposition of fat in the liver, liver sections were stained with Nile red and H&E. As shown in Nile red-stained hepatic sections (Fig. 3), chow-nourished rats did not reveal any accumulation of TG, although fat droplets were observed in the liver sections of the other groups.

**Figure 4:**
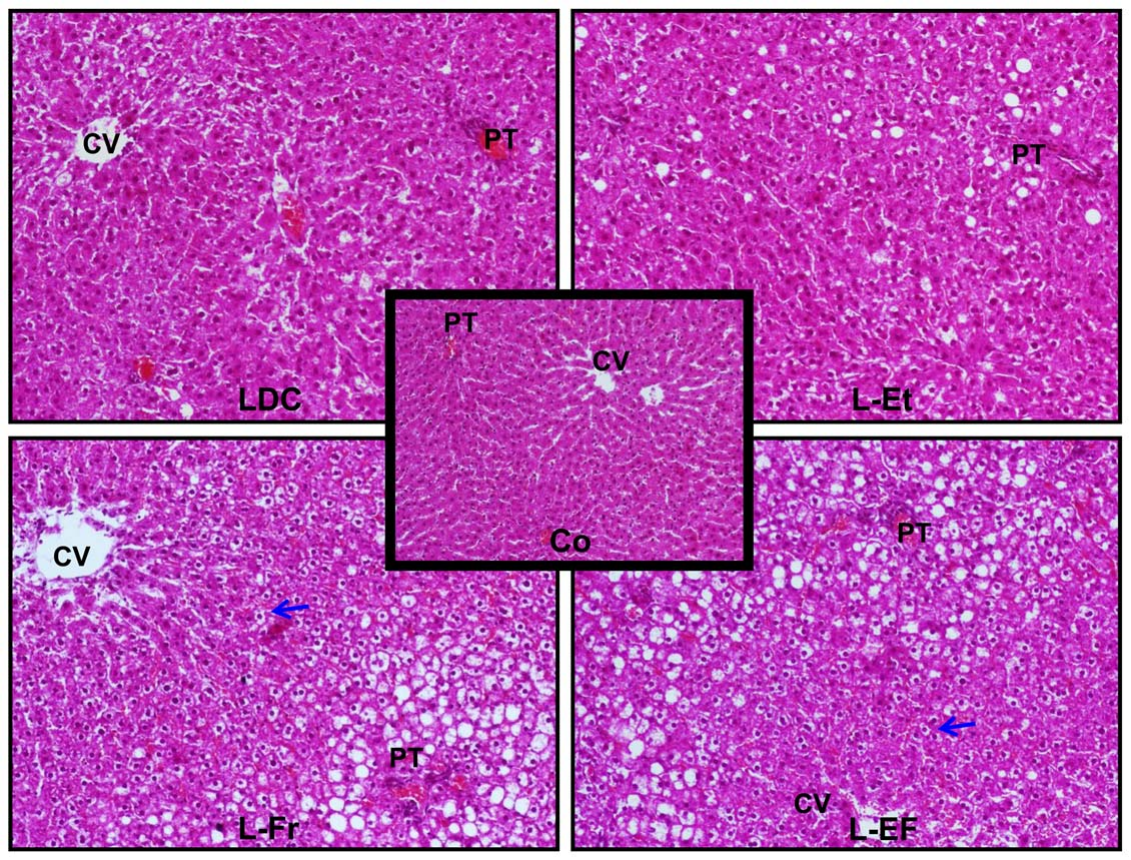
Liver morphology of H&E-stained sections from each rat group after 4 wk of experimental treatment. The LDC-fed animals developed microvesicular steatosis, and there was little or no evidence of inflammation. Both macro- and micro-steatosis were induced by the L-Et diet. In the L-Fr and L-EF regimens, zone I hepatocytes were markedly expanded predominantly by macrovesicular steatosis. Unlike zone I, towards the CV, the fat-loaded hepatocytes have central nuclei (blue arrows). CV: central vein. Original magnification×100.

**Figure 5:**
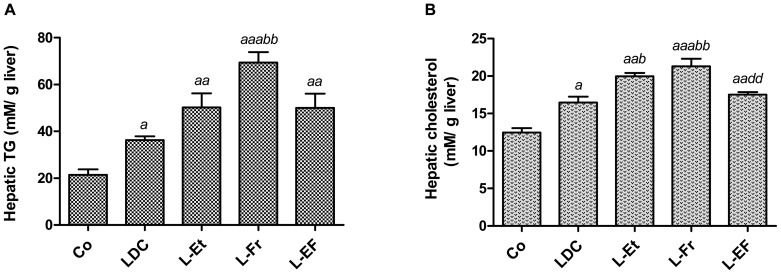
Assessment of intrahepatic TG (A) and cholesterol (B) contents. *^a^*, *^aa^*, *^aaa^*indicates significant difference of the corresponding group *vs*. the Co (*P*<0.05, *P*<0.01, *P*<0.001; respectively), *^b^P*<0.05, *^bb^P*<0.01 *vs*. the LDC group, while *^dd^*denotes *P*<0.01 *vs*. the L-Fr group. The animals (n = 6) were fed for 28 d.

In the control group, a normal lobular pattern and a well-preserved cytoplasm were observed in the hepatic cells, and prominent nuclei were noted in H&E-stained liver sections. No evidence was found for inflammation, steatosis or fibrosis (Fig. 4). At this time point, no inflammation or fibrosis was observed in the livers of LDC-fed rats as well. In the L-Et and L-EF groups, mild portal inflammatory infiltration and stage 1 fibrosis (minimal, mild portal connective tissue) was observed. In addition, both L-Fr and L-EF fed rats exhibited macrosteatosis in the periportal areas. The entire cytoplasm of most hepatocytes in zone I of the hepatic acinus showed steatosis. Different sizes of fat droplets were apparent in the hepatocytes of the L-Et, L-Fr, and L-EF administered animals, whereas the LDC-fed animals mainly showed microvesicular fatty changes with centrally located nuclei (Fig. 4). Inflammation including microgranulomas was also present focally in rats fed L-Fr but not in the other groups (Fig. S1). Lipid accumulation in the experimental groups were further verified by the evaluation of the intrahepatic TG and cholesterol contents (Fig. 5). Compared to the control, hepatic TG and cholesterol levels were increased significantly, 1.8-fold and 1.3-fold (in the LDC group), 2.3-fold and 1.6-fold (in the L-Et group), 3.3-fold and 1.6-fold (in the L-Fr group), and 2.3-fold and 1.4-fold (in the L-EF group), respectively. In addition, the hepatic TG and cholesterol levels in the L-Fr group were higher than those of the LDC group (*P*<0.01).

### Physiological and metabolic changes

#### Liver function test: changes in total plasma protein, albumin, coagulation factors, and the activities of ALT, AST, and AP

In the L-EF and L-Et groups, the total plasma protein concentrations were slightly lower than those of the control group (Table 1). The plasma albumin levels were the lowest in the L–EF group, compared to the other groups (*P*<0.01), indicating a reduction of hepatic synthesis of albumin.

**Table 1:** Plasma biochemical parameters in rats given different diets *ad lib* for 28 d.

	Co	LDC	L-Et	L-Fr	L-EF
Enzymes (U/L)					
ALT	73±5	72±6	82±15	89±9	146±30*^ab^*
AST	148±21	145±19	159±42	465±70*^aabbc^*	382±78*^ab^*
AP	142±10	178±8^a^	203±9^a^	201±12^a^	219±17^a^
Protein (g/dL)	6.1±0.2	6.1±0.1	5.8±0.2	6.2±0.1	5.8±0.1
Albumin (g/dL)	3.9±0.1	4.0±0.1	3.9±0.2	3.9±0.1	3.4±0.1**
PT (Quick)(%)	83±2	80±2	84±2	92±4*^b^*	74±2*^add^*
Urea (mmol/L)	39±3	32±2	26±1*^aa^*	33±2*^c^*	20±2*^aaabd^*
Creatinine (µmol/L)	24.8±0.9	24.7±1.8	25.6±1.8	23.9±1.8	24.8±1.8
Uric acid (µmol/L)	149±24	161±18	208±42	149±24	178±30
Electrolytes (mmol/L)					
Na^+^	148±1	147±2	148±2	146±1	145±2
Cl^−^	102±1	101±2	103±1	103±1	100±1
K^+^	4.6±0.4	4.3±0.3	4.9±0.5	6.4±0.5^a^	5.9±0.3^a^
Fe (µM)	30.9±0.8	33.1±1.9	40.4±2.1^a^	38.1±1.7	44.9±4.1*^aab^*

a , *^aa^*, *^aaa^*denotes statistically significance compared to the Co: *P*<0.05, 0.01, 0.001, respectively. *^b, bb^P*<0.05, 0.01 *vs*. the LDC, *^c^P*<0.05 *vs*. the L-Et, *^d, dd^P*<0.05, 0.01 *vs*. the L-Fr **P<0.01 compared to other groups. Data are presented as mean ± SEM. AP: alkaline phosphatase, ALT: alanine aminotransferase, AST: Aspartate transaminase, Fe: iron. The animals were nourished with: **Co**: control (standard chow diet), **LDC**: liquid Lieber-DeCarli diet **L-Et**: LDC supplemented with 30% J ethanol, **L-Fr**: LDC supplemented with 30% J fructose **L-EF**: LDC+isoenergetic (30%) ethanol and fructose. PT: prothrombin time test.

Another indicator of liver function is the Quick-test, which measures blood coagulation. The coagulation time was significantly longer in the combinational fructose-ethanol fed rats compared to the chow or L-Fr fed rats, pointing to an abnormal synthesis of the clotting factors. Moreover, the activity of SGPT was twofold increased in the L-EF cohort (*P*<0.05) compared to the chow and LDC regimens, while it did not differ among the other groups, as summarized in Table 1. Fructose-containing diets significantly elevated the activity of SGOTcompared with the control and LDC groups. The activity of AP was remarkably elevated in all experimental groups *vs*. control. There were no significant differences in the concentrations of plasma uric acid or bilirubin (data not shown) among the groups. Collectively, the co-administration of fructose and alcohol exaggerates hepatocellular damage.

#### Renal test: plasma creatinine, urea, and electrolyte levels

There were no considerable changes in the concentrations of creatinine, sodium (Na^+^), or chloride (Cl^−^) among the studied groups (Table 1). The highest levels of plasma iron were observed in ethanol-fed rats. On the contrary, the urea levels were the lowest in the ethanol-fed animals and declined to 33% in the L-Et group and 49% in the L-EF group compared to the control (*P*<0.01 and *P*<0.001, respectively). Furthermore, the plasma levels of potassium (K^+^) were approximately 1.4-fold higher in the L-Fr and the L-EF groups (*P* = 0.016).

#### Hematological parameters

The mean values of Hb and erythrocytes were comparable among the groups at day 28 of the experiment (Table 2). The co-administration of ethanol and fructose reduced the platelet count dramatically compared to the other groups. The fructose-given animals showed a significant elevation in the number of leukocytes compared to which given ethanol alone (the L-Et) or the chow.

**Table 2:** Hematological analysis.

	Co	LDC	L-Et	L-Fr	L-EF
Hb (mg/dL)	14.9±0.3	14.7±0.2	14.5±0.3	15.2±0.4	14.8±0.3
Erythrocytes(×10^6^/µL)	7.79±2.71	7.33±3.00	7.57±0.20	7.57±0.36	7.61±0.26
Leukocytes(×10^3^/µL)	6.8±0.1	7.0±0.2	5.4±0.5	9.1±0.6*^acc^*	7.9±0.8*^c^*
Thrombocytes(×10^3^/µL)	699±31	642±8	637±15	765±37*^bc^*	528±12*^aaabcddd^*

EDTA blood was collected from rats after 4 wk of feeding with different diets. *^a, aaa^*indicates statistical significant *vs*. Co: *P*<0.05, 0.001. *^b^P*<0.05 *vs*. LDC, *^c^* ^, *cc*^ *P*<0.05, 0.01 compared to L-Et, while *^ddd^P*<0.001 compared to L-Fr.

#### Plasma lipid profile

**Figure 6:**
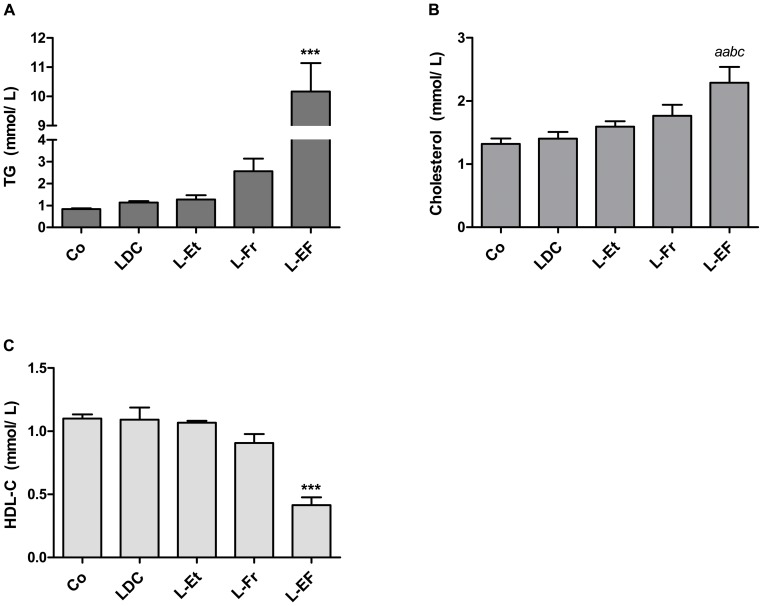
Bar graphs display plasma lipid profile. Rats were given *ad lib* chow, LDC, L-Et, L-Fr or L-EF diet (n = 4). **B**. total cholesterol. *^a^P*<0.01, *^aa^P*<0.05 *vs*. respective the Co. *^b^P*<0.01 *vs*. LDC, while ***P<0.001 compared to all groups. The data are shown as mean and error bars denote SEM.

The ethanol-fructose combined diet significantly worsened the rats’ plasma lipid profiles. The differences between the control and LDC groups were almost negligible. Surprisingly, in the L-EF group plasma TG levels increased 2.7-fold more than the sum of the effects of the L-Et and L-Fr diets individually (Fig. 6A, *P*<0.001). Therefore, the appearance of the plasma in the L-EF-fed rats was creamy (turbid), reflecting the strongly elevated TG levels. The plasma total cholesterol levels were not substantially elevated in the animals nourished with either alcohol or fructose alone compared to controls. In contrast, the administration of the L-EF diet raised the plasma total cholesterol level significantly, which was parallel to the excessive TG levels (Fig. 6B). Contrariwise, the concentration of HDL-cholesterol was reduced (40%) in the L-EF group compared to the control rats (Fig. 6C), and it was the lowest out of all the other experimental groups (*P*<0.001). Still, the levels of LDL-C were almost the same in all groups (data not shown).

#### Changes of glucose, leptin, insulin and C-peptide levels in both fed and fasted rats

**Figure 7:**
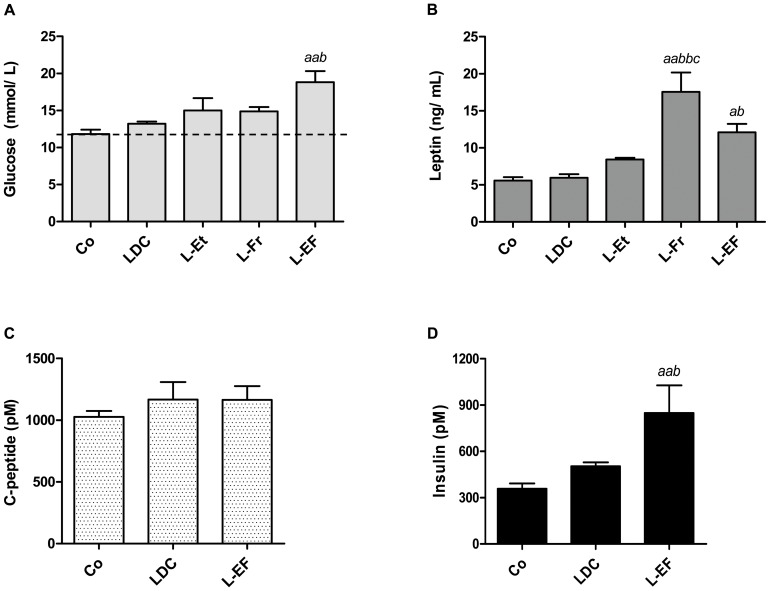
The bar charts show plasma glucose (A), serum leptin (B), C-peptide (C), and insulin (D) concentrations in rats after feeding with assigned diets. The blood was collected from the rats in a fed-state at the end of wk 4. *^a^*, *^aa^*designates the level of significant differences between the corresponding group and the Co (*P*<0.05, *P*<0.01; respectively), *^b^P*<0.05, *^bb^P*<0.01 *vs*. the LDC, *^c^P*<0.05 *vs*. the L-Et.

As presented in Fig. 7A, the blood glucose level was markedly elevated in the L-EF rats fed *ad lib* compared to the chow- or LDC-fed animals. This occurred despite the significantly augmented insulin levels in this group (Fig. 7C). The intake of ethanol or fructose alone did not significantly affect blood glucose levels. In the L-Et and L-Fr groups, glycemia was fairly augmented. Neither the LDC nor L-Et diet significantly up-regulated serum levels of leptin (an appetite and inflammatory regulator) compared to control. However, in fructose-treated animals, serum leptin levels were significantly higher than those in the control or LDC groups (Fig. 7B). The serum C-peptide levels in the LDC and L-EF groups were almost unchanged (Fig. 7C).

As documented previously [15], the fasting plasma glucose level was mildly elevated in the LDC group compared to the controls, whereas the TG level was significantly elevated by 3-fold. Similar to the glucose level, the fasting serum C-peptide and insulin levels were slightly higher among LDC-fed rats (Table 3). The overnight fasted L-EF rats showed a similar profile for their glucose and TG levels and C-peptide:insulin ratio. Plasma TG and glucose levels were significantly augmented by 7.5±0.7 and 1.6±0.3-fold in the L-EF group, compared to the control group and by 3.1±0.4 and 1.3±0.1-fold *vs*. the LDC group (*P*<0.05). No significant changes were observed in the fasting level of C-peptide among the groups, although the serum insulin level was remarkably higher in the L-EF group *vs*. the control or LDC groups (*P*<0.05, Table 3).

**Table 3:** Insulin clearance.

	Co	LDC	L-EF
Insulin (pM)^#^	155±17	190±45	380±33*^aab^*
C-peptide: insulin			
Fasted	4.35±0.30	4.22±0.57	2.31±0.24*^aab^*
Fed	2.95±0.29	2.35±0.37	1.48±0.20*^aab^*

Serum samples were collected from rats after 4 wk of feeding with various diets.

In these subgroups, rats were overnight fasted or fed at time of sacrifice. *^aa^*indicates statistical significant *v*s. Co: *P*<0.01, *^b^P*<0.05 *v*s. LDC. ^#^fasted levels. To estimate insulin clearance, a ratio of the levels of C-peptide to insulin was calculated.

Because the liver is an important site of insulin clearance [24], we calculated the ratio of the serum levels of C-peptide and insulin to estimate the hepatic insulin extraction. C-peptide is a cleavage product of proinsulin; however, unlike insulin, it is cleared by an insulin receptor-independent mechanism in the kidney [24]. The L-EF group (fed-state) showed a reduction by approximately 50% and 38% in the C-peptide:insulin ratio compared to the chow and LDC regimens, respectively (Table 3). These values indicate a marked decrease in the liver insulin clearance, which mostly explains the hyperinsulinemia. Similar findings were also observed in the fasted-rats (Table 3).

### Changes in the mRNA expression of fat metabolism-related genes and insulin pathway in the rat liver tissues

**Figure 8:**
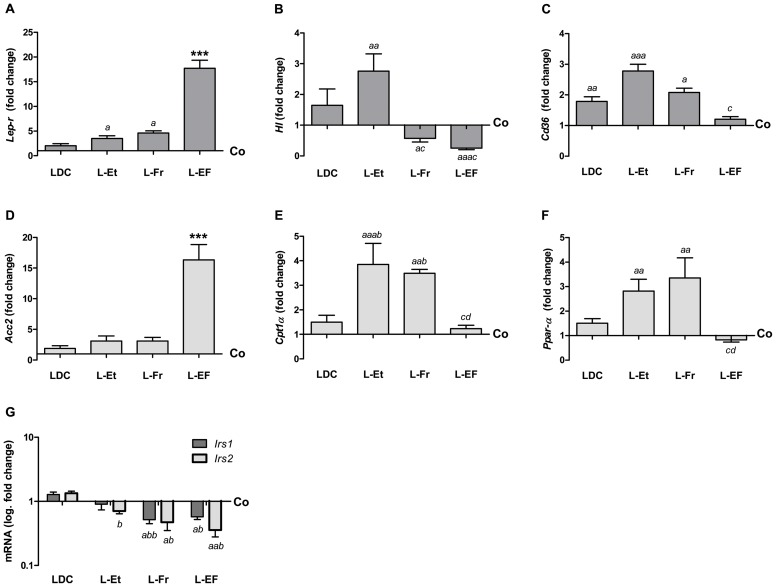
Quantitative analysis of gene transcription in rats fed with different Diets. (I) Bar plots showing the relative levels of (A–F) *Lep-r*, *Hl*, *Cd36*, *Acc2*, *Cpt1a*, and *Pparα* specific mRNA transcripts in hepatic samples from 4 rats/group. PCRs were performed in duplicate. Controls were set as 1 on the y-axis. (G) The diagram shows the fold change in the *Irs-1* and *Irs-2* gene expressions on the logarithmic y-axis *vs*. feeding categories. *Actb* (β-actin) and *Ubc* were used as an internal control. *Lep-r*: leptin receptor, *Hl*: hepatic lipase, *Cd36*: fatty acid transporter, *Acc2*: acetyl-CoA carboxylase 2, *Cpt1a*: carnitine palmitoyltransferase Iα, *Irs*: insulin receptor substrate. *^a^P*<0.05, *^aa^P*<0.01, *^aaa^P*<0.001 *vs*. Co, while ^b^ *P*<0.05 *^bb^P*<0.01 *vs*. LDC, *^c^P*<0.05 *vs*. L-Et, *^d^P*<0.05 *vs*. L-Fr, ***P<0.001 compared to the remaining groups.

Genes encoding key enzymes/receptors were studied. The highest increase in the *Lep-r* transcripts was found in the L-EF group (*P*<0.001). An increase was also found in the L-Fr and L-Et groups (*P*<0.05) compared to the control (Fig. 8A). Hepatic lipase (HL) is a lipolytic enzyme that hydrolyzes TG and phospholipids from the circulating lipoproteins [25]. The hepatic mRNA transcription of *Hl* was significantly up-regulated in the L-Et nourished rats *vs*. the control, L-Fr or L-EF groups; still, these values were substantially down-regulated in the L-Fr (to 43%) and L-EF (75%) groups *vs*. control (Fig. 8B). The maximal transcriptional levels of hepatic *Cd36* (long chain fatty acids uptake) were 2.8-fold (L-Et), 1.8-fold (LDC) and 1.5-fold (L-Fr) *vs*. controls (Fig. 8C). A significant difference in the hepatic *Cd36* expression was noted between the L-Et and L-EF-fed animals. Intriguingly, acetyl-CoA carboxylase (*Acc2,* regulates generation of malonyl-CoA; a substrate for lipogenesis as well as a negative modulator of mitochondrial fat oxidation) transcripts were increased in the liver tissues of L-EF-fed rats compared to all other groups (*P*<0.001).

We also determined a measurable impact of an ethanol and/or fructose-containing LDC diet on hepatic transcription of key function genes in mitochondrial long chain fatty acid β-oxidation, including peroxisome proliferator-activated receptor alpha (PPAR-α) and its downstream target carnitine palmitoyltransferase 1α (*Cpt1 α,* the rate-limiting enzyme).

In both the L-Et and L-Fr regimens, the hepatic expression of *Cpt1α* and *Ppar-α* was induced to a greater extent than in the other groups (Fig. 8E, F). No significant change was observed in the liver tissue of L-EF or LDC-fed rats compared to the control. Quantitative analyses of the mRNA in liver tissues revealed that the co-administration of LDC with either the fructose (L-Fr) or fructose and ethanol (L-EF) diet caused a significant decrease in the levels of *Irs-1* (45% reduction in both groups) and *Irs-2* by (53% in L-Fr and 62% in the L-EF group) compared to the control or LDC group (Fig. 8G). These findings emphasize the unfavorable effects of dietary fructose on IR-accompanied fatty liver.

## Discussion

The ingestion of fast food, a Western-style diet, soft drinks, and/or alcoholic beverages increases in parallel to the increased epidemiology of fatty liver. To characterize the effect and interaction of various diets, we compared the ability of chow, LDC (high fat), L-Et (high fat and ethanol), L-Fr (high fat and high fructose (Western diet)), and L-EF (moderate fat, high fructose, and ethanol) diet to induce hepatic steatosis and damage and to develop a metabolic syndrome in rats fed for 4 weeks. These diets were selected as they represent some of the current most popular food. The various diets’ ingredients are within the range of physiological intake. In this novel study, we found differences in the effects of the long-term consumption of fructose, ethanol or both on animals’ phenotypes.

Parameters, including food intake and BW, were examined in this study. The lowest levels of the rats’ food consumption and BW gain were observed in the first 3 weeks of ethanol administration. It has been reported that the reduction of the rats’ BW by alcohol occurs mainly through a decrease in the meal size and is mediated, in part, by a postgastric mechanism, such as leptin [26], and may be due to a limited intake of the other nutrients as in the L-EF group. We also found that the gene encoding *Lep-r* was up-regulated in liver tissues of the L-Et- and L-EF-nourished animals. However, we cannot exclude an ethanol effect on the hepatic leptin receptors: affecting the receptor itself or its sensitivity to leptin [26]. The addition of fructose to alcoholic HFD did not counteract the suppression of food consumption and the BW mediated by ethanol. Thus the fructose effect can be estimated as minor in this context. However, this effect might be reversed by leptin insensitivity.

### Metabolic syndrome, liver function, and insulin clearance

Notably, we found that the paired administration of ethanol and fructose in a HFD induced an increase in the fasted and non-fasted blood glucose, TG, and insulin levels. In addition, the HDL-C plasma levels were significantly reduced in L-EF-fed rats. The total cholesterol levels were substantially increased, although hypercholesterolemia is unusual without feeding with a cholesterol-enriched diet [5]. This constellation of hyperglycemia, dyslipidemia, hyperinsulinemia [27], together with a fatty liver, has been demonstrated to be associated with IR and metabolic syndrome [28]. These effects collectively confer an increased risk of cardiovascular disease [29].

Hypertriglyceridemia is the most common lipid disorder in type 2 diabetes mellitus (DMII), and its pathogenesis is incompletely understood. This syndrome may result from the increased production and/or decreased clearance of TG-rich lipoproteins [30]. Van de Wiel [17] stated that alcohol taken with a meal leads to an increase in postprandial lipidemia, due to an increase in VLDL synthesis in the liver. Significant declines in the activity of HL may be another reason for hypertriglyceridemia [31]. Indeed, pair-feeding with L-EF or L-Fr significantly decreased the expression of *Hl*. However, the L-Et diet did not significantly increase the plasma TG level compared to any of the other diets. Only the L-EF diet excessively increased plasma TG and reduced the HDL-C levels. Alcohol appears to increase the metabolic rate, causing more energy burn than storage in the body as fat in adipose tissue [32]. In reality, people eat meals consisting of a variety of foods with complex combinations of nutrients that may be synergistic [33]. Thus, the case of dyslipidemia may be attributed to a synergistic interaction between fructose and alcohol in the L-EF group.

**Figure 9:**
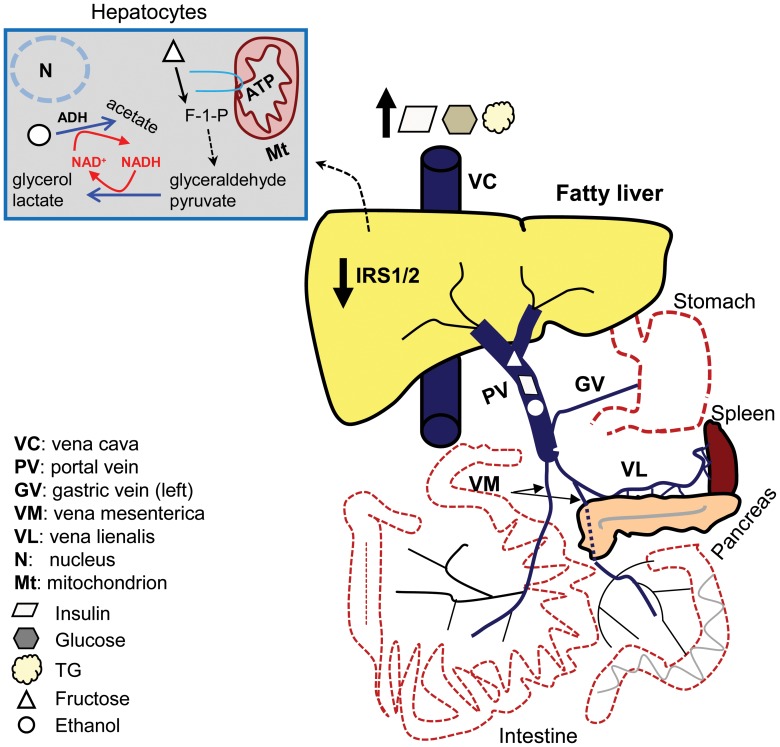
Scheme illustrating the interaction between fructose and ethanol ingested simultaneously to elicit metabolic imbalance. The portal vein delivers the secreted insulin in addition to approximately 80% of the ingested ethanol and most of the fructose and other nutrients to the liver. Fructose and ethanol provide the metabolites that serve as the substrates for *de novo* lipogenesis. Fructose catabolism requires ATP to form F-1-P, glyceraldehyde, and pyruvate. The subsequent process of reducing these metabolites yields NAD^+^, a necessary coenzyme required in alcohol oxidation by ADH. An uncontrolled NADH/NAD^+^ ratio could affect mitochondrial functions. Both nutrients also induce JNK-1 activation, phosphorylating hepatic IRS-1/2 and rendering it inactive while contributing to hepatic insulin resistance. The last effect promotes hyperinsulinemia and influences the substrate’s deposition into fat, promoting hepatic steatosis, gluconeogenesis, and hyperglycemia. The liver is the major site of insulin clearance and has a first-pass clearance above 75%. *Tna-α* (a pro-apoptotic mediator) is expressed in such steatosic livers. ADH: alcohol dehydrogenase, IRS-1/2: insulin receptor substrate-1/2, JNK-1: c-jun N-terminal kinase, F-1-P: fructose-1-phosphate.

Liver function was assessed by measuring prothrombin time, plasma levels of albumin and liver enzymes. The concurrent intake of ethanol and fructose decreased albumin levels, increased insulin and transaminases, and prolonged the prothrombin time, in line with other studies [34]. These functional changes in the livers of L-EF group were associated with a declined C-peptide:insulin ratio, reduced hepatic expression of *Irs-1* and -*2*, and an increased LW. The L-EF fed rats may experience a reversible reduction of hepatocellular function (including reduction of the insulin-signaling pathway-proteins and the reduction of insulin and glucose uptake by the hepatocyte) [35] without experiencing a reduction in the total cell number. This could be due a joint negative effect of fructose and ethanol on the insulin and glucose pathways. Indeed, fructose and ethanol provide metabolites that serve as substrates for *de novo* lipogenesis in the liver. They also induce JNK-1 activation, which phosphorylates hepatic IRS-1/2, rendering them inactive [36]. This action also contributes to hepatic IR and hyperinsulinemia because the liver is major site of insulin clearance and the first pass clearance is above 75% [24], [35], as depicted in Fig. 9. Of note, there is still a caveat about C-peptide and insulin’s half-lives from 30 years ago [37]. IR in DMII is more frequently detected in patients [38] and animals [39] with NAFLD, both, with or without classic obesity.

An earlier *in vitro* study showed that a synergistic interaction between fructose and ethanol promotes hepatocyte sensitivity to TNFα-induced necroptosis via a mitochondrial 2Fe-2S-containing protein [40]. However, this effect was reversed by the pretreatment of the cells with an anti-diabetes agent (pioglitazone) [40]. Taken together, our findings have demonstrated that the combination of alcohol and fructose is able to trigger hepatic IR and diabetes, as well as liver dysfunction in the absence of an increased BW, mostly via synergistic interaction.

### Consumption of alcohol, fructose, or both and fatty liver

We found that all diets except the control induced heaptic steatosis. The LDC-fed rats developed hepatosteatosis, which had resulted from their increasing exogenous fat load. The highest hepatic TG content was measured in the L-Fr group. We previously fed rats with LDC +70% kcal fructose for 4 or 8 weeks. An elevated fasting plasma TG level and a massive hepatic macrosteatosis were observed in this model [15]. In contrast, the L-EF diet showed less steatosis but more increase of the plasma TG levels. The L-EF diet also induced an uneven distribution of fat droplets in the portal and centrilobular areas. Hepatic mRNA expression studies highlighted three potential mechanisms by which feeding with L-EF can lead to increased hepatic steatosis: there was no increase in β-oxidation of fatty acids (e.g., *Ppar-a* and *Cpt1α*) compared to control, despite of fat ingestion; increased *de novo* lipogenesis (e.g., *Acc2*); and reduced *Irs-1* and *-2* expression. Our results are consistent with those of previous studies [41] in suggesting that PPAR-α functions as a sensor for fatty acids and that ineffective PPAR-α sensing can lead to reduced energy burning and inadequate β-oxidation, resulting in hepatic steatosis. Fiebig and colleagues [42] demonstrated that high-fructose feeding increases *de novo* lipogenesis by up-regulating the hepatic lipogenic enzymes, e.g., ACC2 [43]. It is unclear how (hepatic) IR facilitates increased liver glucose production, whereas the responsiveness of lipogenesis to insulin is not affected. The suppression of insulin-signaling pathways leads to the uncontrolled activation of hepatic SREBP1 c, which in turn results in fatty liver [44]. In the L-Fr and L-EF groups, where we found a reduction of *Irs-1/2* transcripts in the liver, a significant elevation of the leukocyte counts was also observed compared to the controls. Likewise, other studies have documented that IR is often associated with chronic low-grade inflammation [45].

### Impact of fructose and ethanol metabolism on oxidative stress and mitochondrial impairment

It was shown that fructose underwent a *Maillard* reaction, promoting lipid peroxidation and mitochondrial damage, and decreasing the mitochondrial-biogenic proteins, e.g., PGC1α in the liver [15], [36]. Similar effects were caused by ethanol consumption in the decreasing hepatic PGC1α [46], and triggering reactive oxygen species (ROS) production and iron overload in the liver [47]. In fact, Zhong *et al*. found that ethanol could induce hepatic mitochondrial dysfunction (affecting the mtDNA by forming protein adducts, which impair ATP production). It also causes a deficiency of Cyp2E1, that is responsible for ethanol metabolism [48]. Although a great deal of data have been published about the effects of fructose and ethanol individually on the hepatocellular metabolism, the chronic effects of both compounds have not been thoroughly elucidated. Lustig [36] has reported that both hepatic metabolisms are similar, as both induce lipogenesis and the fructosylation of proteins, resulting in superoxide formation, similar to acetaldehyde (a metabolite of ethanol). In our study, co-administration of ethanol and fructose resulted in a mild portal inflammation and fibrosis. This combined diet also increased markedly the hepatic expression of metabolic and inflammatory regulators [49] e.g., lipocalin-2 and melanocortin 4 receptor compared to each cohort alone (data not shown). Indeed, we [15], [50] and others [51] have found that administrating high-fructose, FeCl3, or ethanol induced serum and/or hepatic expression of lipocalin-2, an acute-phase protein that is sustainably expressed in response to oxidative stress conditions. The hepatic expression of *Tnf-α* (a pro-apoptotic mediator) was also increased in the fatty livers. In overall, these effects imply that the ROS production and mitochondrial dysfunction may be magnified by the long-term, jointly ingestion of an ethanol and fructose.

In fact, the short-term interaction between ethanol and fructose has been clinically investigated, as well. The administration of fructose, taken with alcohol drinking, reduced acute ethanol intoxication [52] by accelerating its metabolism and elimination rate from the bloodstream [53], support an antagonistic interaction. The catabolism of ethanol to acetaldehyde is mainly catalysed by alcohol dehydrogenase (ADH), coinciding with a reduction of NAD^+^ to NADH. Actually, the overall rate of alcohol oxidation is limited by the rate of NAD^+^ generation [53]. Although we did not measure the NADH/NAD^+^ ratio in our study, these values are also responsible for steatosis by inhibiting β-oxidation and promoting fatty acid synthesis [48]. Interestingly, during fructose catabolism the formed glyceraldehyde and pyruvate undergo a reduction reaction, the process that includes the reoxidation of the coenzyme NADH to NAD^+^ (Fig. 9). Although large doses of fructose are likely to be risky for metabolic acidosis [36], a concomitant intake of ethanol increases this risk [52].

The liver is the main site of alcohol oxidation. When all other known causes of steatosis have been excluded, the cut-off level for what is considered to be tolerable alcohol consumption ranges from abstinence [54] to, most commonly, 30 g/day, which is used as a threshold to differentiate between AFLD and NAFLD [8], [55], otherwise, it is regarded as a heavy consumption [56]. This seems to be a reasonable definition when evaluating patients with suspected NAFLD in clinical practice [55] and increases the understanding of NAFLD and ALD pathogeneses and of their clinical overlap. In our study, rats were given approximately 5.8% *(v/v)* alcoholic diets, similar to another study in which light alcohol (6% *v/v*) solution and pelleted diet were given chronically for 4 weeks [57]. Such a dose of alcohol, when combined with fructose, led to significant adverse impacts on the liver and systemic biochemical parameters. Because the amount of alcohol consumed can only be documented according to self-reports, this procedure often underestimates the real quantity consumed. *Kechagias et al*. have stated that in their clinical evaluations of subjects with elevated ALT levels, medical histories should include not only questions about the alcohol and soft drink intake but should also explore whether recent excessive intakes of fast food had occurred [11].

Together, a marked reduction in the rats’ weight gain was mainly induced by ethanol, which is suggestive of a minor impact of adding fructose to ethanol on the BW at this time point. Although both diets led to the development of hepatic steatosis, the fructose-enriched diet induced more metabolic disturbances, in total, than equivalent calories derived from ethanol-enriched diet. The catabolism of fructose and ethanol administered together yielded metabolites that may contribute to oxidative stress and mitochondrial impairment. Furthermore, the combined ethanol-fructose diet not only induced a level of reduction in protein synthesis but also exacerbated dyslipidemia and DMII, both of which are risk factors for cardiovascular disease. These clinically relevant models indicate the significance of certain nutrients in inducing undesirable adverse effects on metabolic syndrome and liver function, especially on insulin elimination and glucose and lipid metabolism. Therefore, therapeutic approaches should focus primarily on the lifestyle modification.

## Supporting Information

Figure S1 **Micrographs (A) ×100 and (B) ×400 of H&E stained liver sections of L-Fr fed rats.** Blue circles mark microgranulomas.
(TIF)

Table S1 **Composition of Lieber-DeCarli powder.**
(DOC)
