# Supplementary figures and images for: Combination of Alcohol and Fructose Exacerbates Metabolic Imbalance in Terms of Hepatic Damage, Dyslipidemia, and Insulin Resistance in Rats
Source: PLoS One. 2014 Aug 7;9(8):e104220. doi: 10.1371/journal.pone.0104220 (PMC4125190; doi:10.1371/journal.pone.0104220)

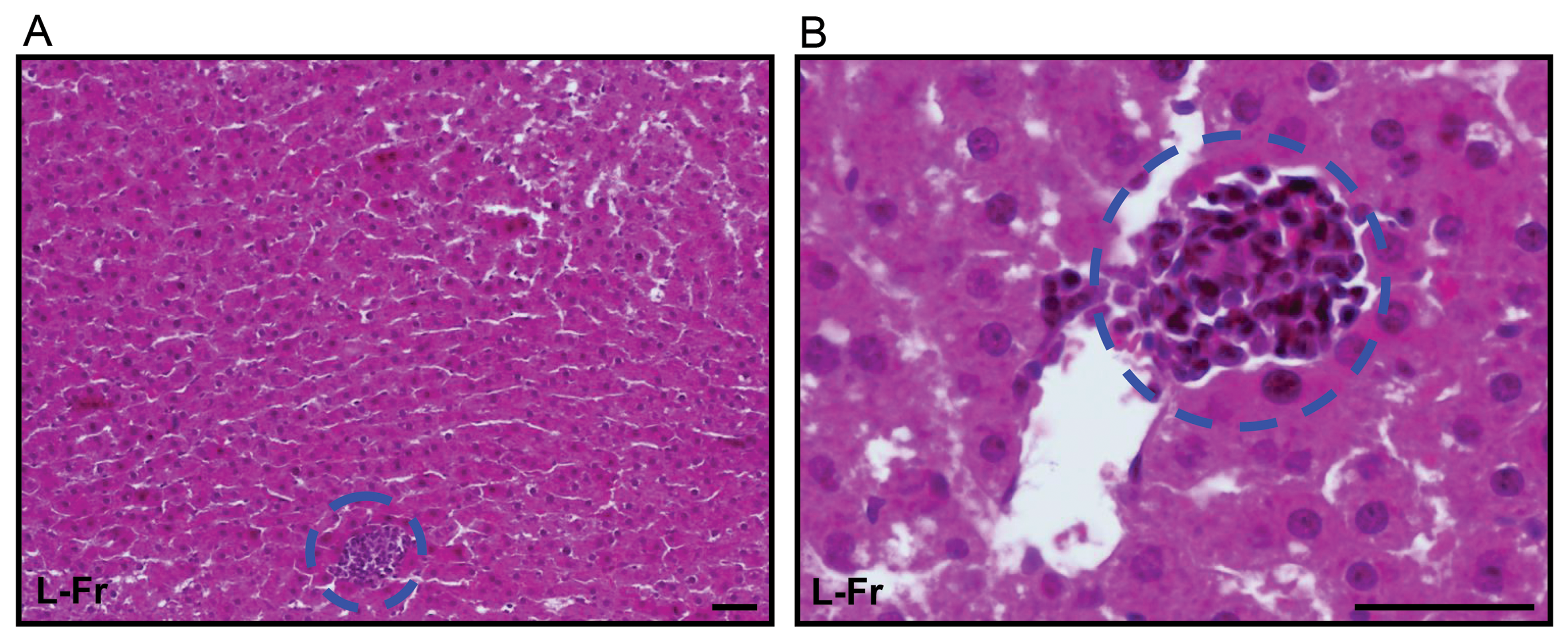

Supplement: Figure S1 — Micrographs (A) ×100 and (B) ×400 of H&E stained liver sections of L-Fr fed rats. Blue circles mark microgranulomas. (TIF) [file pone.0104220.s001.tif]
