# Supplementary material for: Combination of Alcohol and Fructose Exacerbates Metabolic Imbalance in Terms of Hepatic Damage, Dyslipidemia, and Insulin Resistance in Rats
Source: PLoS One. 2014 Aug 7;9(8):e104220. doi: 10.1371/journal.pone.0104220 (PMC4125190; doi:10.1371/journal.pone.0104220)
Supplement: Table S1 — Composition of Lieber-DeCarli powder. (DOC) [file pone.0104220.s002.doc]

**Table S1**

**Composition of Lieber-DeCarli powder.**

| **Crude nutrients ^1^** | **Percentage** |
| --- | --- |
| Protein | 16.5 |
| Fat | 18.0 |
| Carbohydrates (N free extracts) | 55.9 |
| Maltodextrin | 51.4 |
| Sugar | 1.5 |
| Fiber | 4.5 |
| Ash | 3.0 |

^1^ The diet contains also micronutrients like amino acids, vitamins, minerals, trace elements, and un-/saturated fatty acids. The LDC was provided by ssniff Spezialdiäten GmbH, Soest, Germany.
